# Pediatric Non-Down Syndrome Acute Megakaryoblastic Leukemia Patients Have Dismal Outcomes Irrespective of Allogeneic Hematopoietic Stem Cell Transplant: A Single-Center Experience

**DOI:** 10.3390/cancers17213511

**Published:** 2025-10-31

**Authors:** Gabriela Llaurador, Matthew Willis, Michele S. Redell, M. Monica Gramatges, Andrea N. Marcogliese, Swati Naik, Robert Krance, Erin Doherty, Alexandra M. Stevens

**Affiliations:** 1 Department of Bone Marrow Transplantation, Texas Children’s Cancer and Hematology Center, Baylor College of Medicine, Houston, TX 77030, USA; 2 Baylor College of Medicine, Texas Children’s Cancer and Hematology Center, Texas Children’s Hospital, Houston, TX 77030, USAmlredell@texaschildrens.org (M.S.R.);; 3 Department of Pathology, Baylor College of Medicine, Texas Children’s Hospital, Houston, TX 77030, USA; 4 Department of Bone Marrow Transplantation and Cellular Therapy, St. Jude Children’s Research Hospital, Memphis, TN 38105, USA

**Keywords:** AMKL, AML, myeloid leukemia, relapsed leukemia, acute megakaryoblastic leukemia, bone marrow transplant, hematopoietic stem cell transplant

## Abstract

**Simple Summary:**

Pediatric acute myeloid leukemia (AML) is a challenging disease to treat. Patients with relapsed or refractory disease have poor overall survival outcomes despite aggressive chemotherapy and hematopoietic stem cell transplant. This is particularly true for non-Down syndrome patients with an aggressive subclassification of AML: acute megakaryoblastic leukemia (AMKL). While only comprising a fraction of total pediatric AML diagnoses, this disease subtype has among the highest mortality rates. Our research sought to identify trends in the outcomes at a single, large, tertiary care hospital. Outcomes were suboptimal despite the use of allogeneic stem cell transplantation in first remission, with disease relapse being the main cause of mortality in our cohort. Results from this study demonstrate the need for continued research on novel drug development designed to treat this high-risk subtype, with expedient translation to the bedside.

**Abstract:**

Background: Pediatric non-Down Syndrome Acute Megakaryoblastic Leukemia (non-DS-AMKL) is a rare subtype of Acute Myeloid Leukemia (AML) arising from primitive megakaryocytes and is associated with poor outcomes. Given its high incidence of relapse, this subpopulation of children is frequently referred for allogeneic hematopoietic stem cell transplant (allo-HSCT) in first complete remission (CR1). Objectives: The objective of this study was to describe the clinical outcomes of non-DS-AMKL pediatric patients in a large, single-institution cohort. Methods: A retrospective review of the medical records of thirty-six patients diagnosed with non-DS-AMKL treated at Texas Children’s Hospital from 2000 to 2022 was conducted. Results: Twenty-nine patients were included in the analysis, with cohorts defined by intention to treat. Twelve patients received chemotherapy only during upfront therapy, and seventeen received upfront HSCT. The 5-year overall survival (OS) and disease-free survival (DFS) for the entire cohort were 19.1% and 24.1%, respectively, with a median survival of 17.4 months. A higher percentage of patients in the chemotherapy-only group had relapsed/refractory disease at death (chemotherapy only, *n* = 9; HSCT, *n* = 8). However, 5-year OS and DFS were similar for both groups (OS = 18.8% vs. 31.3%, *p* = 0.58; DFS = 37.6% vs. 22.2%, *p* = 0.51). Relapse was the leading cause of death (5-year cumulative incidence of relapse (CIR) 0.78). Treatment with allo-HSCT did not improve outcomes due to the high CIR, even after HSCT in CR1. Conclusions: These dismal outcomes highlight the need for development and incorporation of novel targeted agents into upfront therapy or in the post-HSCT setting for patients with this challenging disease.

## 1. Introduction

Acute Megakaryoblastic Leukemia (AMKL) is a subtype (FAB-M7) of Acute Myeloid Leukemia (AML) arising from primitive megakaryocytes. AMKL, while the most common subtype of AML in children with Down Syndrome (DS), accounts for only 3–10% of AML in children without DS [1,2,3]. It commonly presents as pancytopenia, bone pain, or symptomatic anemia and has proven to be challenging to diagnose due to the low percentages of blast cells and characteristic fibrosis of the bone marrow (BM) [4]. Diagnosis has been refined with improvements in immunohistochemistry and immunophenotyping by flow cytometry. Characteristic diagnostic features of AMKL include megakaryoblasts that stain positive for CD41a, CD42, CD61, and are typically negative for MPO. A significant percentage of patients with AMKL also express the RAM phenotype, an aggressive subtype, characterized as CD56+, CD38 and CD45 dim-to-negative, and by a lack of HLA-DR expression [5]. Cytogenetic mutations and karyotype abnormalities in non-DS-AMKL are heterogeneous, but often include complex karyotypes, with recurrent oncogenic translocations such as CBFA2T3::GLIS2, NUP98::KDM5A, RBM15::MKL1, and KMT2A gene rearrangements [6,7].

Non-DS-AMKL represents an aggressive disease with significantly worse outcomes compared to DS-AMKL (10–70% vs. 70–95% overall survival (OS)) [3,8,9,10,11]. Survival rates in non-DS-AMKL patients are impacted by specific prognostic factors. Poor prognostic indicators include the following: RAM phenotype, NUP98::KDM5A, CBFA2T3::GLIS2, and KMT2A–rearrangements. While some groups have reported improved outcomes after intensification of induction treatment, relapse rates remain high (31–53%) [1,10,12,13,14,15,16]. A large multicenter study (*n* = 153) from 2016 demonstrated a 4-year OS of 56%, 4-year event-free survival (EFS) of 51%, and a 4-year cumulative incidence of relapse (CIR) of 29%, notably worse compared to other AML subtypes [17]. Given the historically poor outcomes for this disease, patients with poor prognostic genetic aberrations are frequently offered an allogeneic hematopoietic stem cell transplant (allo-HSCT) in first complete remission (CR1), if a donor is available [18]. The rationale for an approach to treatment that includes allo-HSCT is to optimize post-remission ongoing treatment by leveraging the graft-versus-leukemia effect. However, allo-HSCT is associated with increased risk of treatment-related mortality (TRM) due to intensive conditioning regimens and transplant-related complications.

Evidence of the benefit of allo-HSCT in improving outcomes for non-DS-AMKL is sparse and conflicting, with few studies that compare allo-HSCT to chemotherapy alone [15]. It is noteworthy that, due to the rarity of this condition and sparse outcome data, a diagnosis of non-DS-AMKL by morphology is not currently considered a high-risk feature in the most recent Children’s Oncology Group (COG) risk stratification schema [19]. As a result, some clinicians, relying on personal experience, may elect to deviate from protocol therapy and include upfront allo-HSCT, thus further contributing to the dearth of outcome data for this disease. An analysis of real-world data may facilitate a better understanding of the impact of HSCT in non-DS-AMKL clinical outcomes. Here, we investigated the outcomes of pediatric patients with non-DS-AMKL in a retrospective cohort analysis, according to the intention to treat with upfront therapy that included either chemotherapy alone or chemotherapy and allo-HSCT at a single institution, comparing OS, DFS, CIR, and TRM between the groups.

## 2. Materials and Methods

### 2.1. Study Design and Inclusion Criteria

This study was approved by the Baylor College of Medicine Institutional Review Board. Patients diagnosed with non-DS-AMKL were identified by data obtained by the Center for International Bone Marrow Transplant Research database and patient lists maintained by the clinical teams. The medical records of 36 patients with non-DS-AMKL treated with chemotherapy alone or with chemotherapy and allo-HSCT at Texas Children’s Hospital from 2000 to 2022 were reviewed. All patients had a confirmed diagnosis of non-DS-AMKL by pathology review. Patients were excluded if they had received HSCT or treatment for non-DS-AMKL prior to our study timeframe. All patients were treated with induction I and induction II before treatment divergence to either chemotherapy alone or proceeding towards allo-HSCT for upfront therapy. Patients were assigned to comparison groups (chemotherapy alone vs. chemotherapy plus allo-HSCT) after treatment divergence. Patients who received more than one allo-HSCT were included but were censored at the date of second HSCT for DFS analysis as a measure of evaluating the outcome and success of the first transplant.

### 2.2. Patient and Disease Characteristics

Patients were enrolled or treated according to Pediatric Oncology Group or COG AML protocols based on eligibility criteria or at the discretion of their oncologist. Bone marrow aspirate and biopsy and diagnostic lumbar puncture for cerebrospinal fluid (CSF) analysis were performed for all patients at diagnosis. Diagnosis was confirmed by BM morphology, flow cytometry, and cytogenetic testing. For uniformity, risk stratification was assigned based on COG AAML1031 risk stratification, with the inclusion of RAM phenotype patients in the high-risk group when data were available. Molecular testing was used to assess genetic alterations or fusions such as KMT2A-rearrangements and CBFA2T3::GLIS2 (starting in 2017, when relevant probes became available). Disease status was assessed after each chemotherapy cycle. Patients were considered as being in morphologic CR if <5% leukemic blasts were detected in the BM by morphology. Minimal/measurable residual disease (MRD) assessment by multiparameter flow cytometry was performed in BM aspirate samples after each cycle. MRD+ status was defined as ≥0.1% myeloid blasts detected, based on the AAML1031 protocol definition.

Patients were referred to allo-HSCT by their oncologist for relapsed or refractory disease, high-risk disease status based on MRD+ at end of induction, the presence of high-risk cytogenetic or phenotypic abnormalities, or at the physician’s discretion. Bone marrow and CSF studies were repeated after the last cycle of chemotherapy and prior to HSCT to assess disease status. Disease status prior to HSCT was defined as MRD+ or MRD−, and as CR1, CR2 (second complete remission after relapse), or active (morphologic evidence of disease with ≥5% of blasts in the BM or detection of disease at any site). Bone marrow and CSF analysis were repeated on day +30 and +100 post-HSCT to assess for disease relapse, and at additional time points at the discretion of the treating clinician.

### 2.3. Donor, Graft, and Transplant Conditioning Regimen Characteristics

Patients who underwent allo-HSCT received stem cells from human leukocyte antigen (HLA)-matched or mismatched related or unrelated donors. Matched sibling donors (MSD) with a 10/10 loci match were preferentially used as donors. If an MSD was unavailable, other donors were considered, including 10/10 matched unrelated donors (MUD) or mismatched unrelated donors (MMUD) identified through the National Marrow Donor Program or haplo-identical family members (5 to 8/10 loci matches). Stem cell sources included BM, peripheral blood stem cells (PBSCs), or umbilical cord blood (UCB). A minimum of 5/6 loci match at HLA-A, -B, and -DR and more than 5 × 10^7^ cells/kg of recipient body weight were required when UCB was used as stem cell source.

Patients received one of three types of myeloablative conditioning regimens at the discretion of the treating physician: busulfan/cyclophosphamide-based with or without fludarabine, total body irradiation (TBI) in combination with cytarabine and cyclophosphamide, or busulfan, thiotepa, and fludarabine in combination with rituximab. Specific details on the conditioning regimens are presented in the supplemental materials.

### 2.4. Supportive Care

Graft-versus-host disease (GVHD) prophylaxis consisted of a calcineurin inhibitor with or without methotrexate and additional serotherapy with alemtuzumab or anti-thymocyte globulin for all patients receiving HSCT from MUD, MMUD, and haplo-identical donors. Mycophenolate and cyclosporine were used for all UCB recipients. T-cell depletion with CD34+ selection was used for all haplo-identical donor PBSC/BM recipients while post-transplant cyclophosphamide was used for one haplo-identical donor BM recipient. Institutional guidelines were followed for supportive care, including blood product transfusions, antimicrobial prophylaxis, and supportive medications.

### 2.5. Study Endpoints

The primary endpoints were 5-year OS, DFS, and event-free survival (EFS). Overall survival was assessed from the time of diagnosis to last follow-up or death. DFS was defined as survival without evidence of relapsed disease. EFS was defined as time from diagnosis to any event, including induction failure, relapse, death, or subsequent stem cell transplant secondary to toxicity. As part of the survival analysis for transplant recipients, DFS measurements for patients who received a transplant for relapsed disease started from the time of transplant to a qualifying event. Secondary endpoints included CIR after chemotherapy and/or HSCT, cumulative incidence of TRM and incidence of acute and chronic GVHD.

### 2.6. Definitions

Neutrophil engraftment was defined as an absolute neutrophil count >500/µL for three consecutive days or >1000/µL for two consecutive days. Relapse was defined by morphological, cytogenetic, or flow cytometric evidence of disease at any site (peripheral blood, BM, CSF, or localized chloroma). Treatment-related mortality was defined as death due to any cause other than disease and while in CR. Acute and chronic GVHD were defined using standard Glucksberg criteria [20].

### 2.7. Statistics

Descriptive statistics were used to summarize patient, disease, and transplant characteristics. Overall survival, DFS, CIR, and TRM were analyzed using the Kaplan–Meier method and comparisons were performed using the log-rank test. All analyses were performed with Graph Pad Prism, version 9.40. *p*-values < 0.05 were considered statistically significant.

## 3. Results

### 3.1. Patients and Disease Characteristics

Thirty-six patients were included in the study review. Patient assignment into comparison groups (chemotherapy only vs. chemotherapy followed by allo-HSCT) was determined by the intention to treat based on initial diagnosis, morphology, cytogenetics, and response to treatment, with divergence of the groups occurring after completion of two cycles of induction chemotherapy. Several patients were not included in the comparison groups; four patients were censored for refractory disease never achieving remission, two patients died during induction chemotherapy from infectious causes, and one patient was censored due to a prolonged gap in upfront treatment secondary to extraneous social factors. Twelve patients were treated with the intent for chemotherapy only, with five patients later undergoing allo-HSCT for relapsed disease. Seventeen patients received chemotherapy followed by allo-HSCT as part of upfront therapy. Patient and disease characteristics for the comparison groups are detailed in Table 1 (*n* = 29). Most patients in the comparison groups self-identified as Hispanic-White (*n* = 12, 41%), followed by non-Hispanic African American (*n* = 10, 34%) and non-Hispanic White (*n* = 5, 17%). Most cases (55%) were diagnosed between 2010 and 2022. Median age at diagnosis for the comparison cohort was 2 years. Central nervous system (CNS) disease involvement was observed in 17% of patients. Molecular testing was completed in 11 cases and detected CBFA2T3::GLIS2 fusion in 4 patients.

The median age at diagnosis was 2 years for patients treated with chemotherapy alone and 2 years for those who received HSCT (range 0.7–17 and 0.6–15 years, respectively). Over three-fourths of all patients were in the 0-to-5-year age range. Risk stratification significantly differed between groups, with more patients classified as high risk in the chemotherapy and allo-HSCT group (*p* = 0.015). Cytogenetic and molecular findings were diverse across both groups. Two patients in the allo-HSCT group had both KMT2A rearrangements as well as alterations in chromosome 7. Between the groups, KMT2A rearrangements were more common in the chemotherapy plus allo-HSCT cohort (29% vs. 8%). No patients in the allo-HSCT group had normal cytogenetics.

Three patients without HR cytogenetics did not achieve CR after induction I and therefore went on to receive a stem cell transplant. Five patients without HR cytogenetics achieved MRD positive CR1 following induction II, and subsequently received a stem cell transplant. Additionally, five patients without HR cytogenetics who achieved MRD-negative CR after induction I were referred for upfront allo-HSCT based on physician assessment of disease risk. In the chemotherapy only group, three patients were MRD-positive after induction I, with only one having measurable disease after induction II. These patients did not have high-risk cytogenetics and were not considered to have high-risk disease by the treating physicians. It is noteworthy that these three patients were treated according to AAML0531, which did not stratify patients according to treatment response.

### 3.2. Initial Chemotherapy Regimens and Outcomes

**Figure 1:**
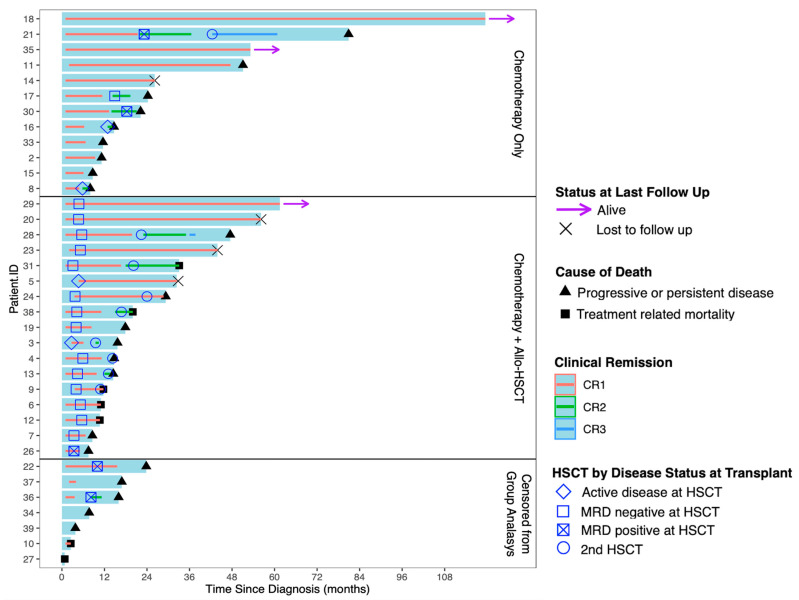
Swimmer plot for chemo only and HSCT groups.

All but three patients were enrolled on or treated according to Pediatric Oncology Group (POG) or COG AML chemotherapy protocols. Patient-level data demonstrating response to therapy and the timeline of their treatment course is seen in Figure 1. Two patients died from infection during induction chemotherapy, and one patient experienced a prolonged lapse in treatment after induction I. Amongst the remaining cohort, 85% (*n* = 28) of patients achieved morphologic CR1 by the end of induction II; however, five of those patients were still MRD positive. Of the four patients who were refractory to induction therapy, three never achieved remission and died with active disease. One patient achieved MRD positive CR1 and received a transplant, before later dying from relapsed disease.

In the chemotherapy + allo-HSCT group, two patients did not achieve morphologic CR at the end of induction II, compared to zero patients in the chemotherapy only group. A higher proportion of patients achieved MRD-CR following induction I treatment in the chemotherapy-only group (*n* = 9, 75% Vs. *n* = 7, 41%). Additionally, 92% (*n* = 11) of patients within the chemotherapy only group achieved MRD- CR by the end of induction II, compared to 71% (*n* = 12) of patients treated with chemotherapy + allo-HSCT.

### 3.3. Pre-Transplant, Donor, Stem Cell Source, Graft, and Conditioning Regimen Characteristics

Of all 36 patients in this study, 24 patients received chemotherapy followed by allo-HSCT, including 5 who were in the chemotherapy-only group that relapsed before going on to receive a transplant. The patient and transplant characteristics are summarized in Table 2. Median age at allo-HSCT was 3 years (range: 0.8–19.3 years) with a median of 149 days (range: 83–697 days) from diagnosis to HSCT. Most patients received BM-sourced stem cells from a MUD (*n* = 9, 38%) or a haploidentical donor (*n* = 9, 38%). Fourteen patients were in MRD-negative CR1 by flow cytometry prior to allo-HSCT (58%), with two patients in MRD-negative CR2. Eight patients (22%) had measurable disease, with four of those demonstrating active disease with >5% blasts. The most common indications for allo-HSCT were MRD positivity after induction I (*n* = 10, 28%), or consideration of disease as high-risk (*n* = 9, 25%). The classification of high-risk (HR) disease status was based on HR features (monosomy 7, *n* = 2; 5q deletion, *n* = 1; RAM phenotype, *n* = 1), or consideration as HR by the treating physician (*n* = 5), despite a lack of HR cytogenetics and MRD-negative status at the end of induction.

### 3.4. Survival Outcomes

**Figure 2:**
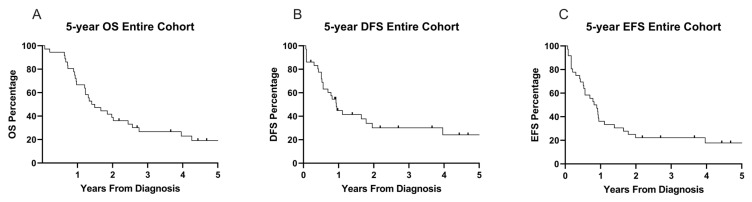
Five-year outcome data for the entire cohort. (**A**) Five-year OS with deaths/events, *n* = 29, and censored patients/survivors, *n* = 7. Patients were censored at the last known follow up. (**B**) Five-year DFS, with relapses/events *n* = 24, and censored patients/disease-free *n* = 12. (**C**) Five-year EFS with relapse, as well as transplant for, and deaths from all causes counted as events.

**Figure 3:**
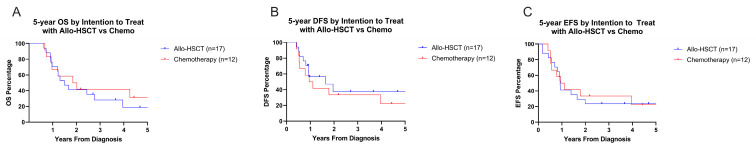
Five-year survivorship data of intention to treat cohorts. (**A**–**C**) Five-year OS and DFS data, respectively, comparing the chemotherapy only group vs. the group treated with allo-HSCT.

Figure 2 summarizes patient outcomes for the entire cohort of 36 patients. The 5-year OS, DFS, and EFS were 19.1%, 24.1%, and 17.8%, respectively (Figure 2A–C), with a median follow up time of 17.4 months from diagnosis (range: 0.8–120 months). When comparing the intention to treat groups, 5-year OS was statistically similar between the groups given the high *p*-value (OS: 18.8% for chemotherapy and allo-HSCT vs. 31.3% for chemotherapy alone, *p* = 0.58; Figure 3A). Comparing 5-year DFS and EFS, there was no difference between the groups (DFS: 37.6% for chemotherapy and allo-HSCT vs. 22.2% for chemotherapy alone, *p* = 0.51, EFS: 23.5% for chemotherapy and allo-HSCT vs. 22.2% for chemotherapy alone, *p* = 0.80; Figure 3B,C). Five-year DFS trended higher in these survival outcomes, likely due to the number of deaths from infection. Overall survival was similar between the two groups at one year (Supplemental Figure S1).

In the chemotherapy-only group, two patients (17%) remain alive in CR1, with an additional patient alive at two years post-diagnosis before being lost to follow up. Nine of the twelve patients in this group developed relapsed disease, five of whom received an allo-HSCT. All nine of the patients who relapsed died from relapsed/refractory disease progression. In the chemotherapy + allo-HSCT group, one patient remains alive, and three additional patients were alive at the last visit date before being lost to follow up. Relapsed/progressive disease was the cause of death for nine patients in the chemotherapy + allo-HSCT group, while the remainder died from viral infection after day 100 post-HSCT (*n* = 3), bacterial and fungal sepsis in the setting of refractory GVHD (*n* = 1), and pulmonary hemorrhage (*n* = 1). Patients who underwent allo-HSCT treatment from 2010 to 2022 had improved OS compared to those who received it from 2000 to 2009 (5-year OS: 2000–2009 = 11.1%, (*n* = 9) vs. 2010–2022 = 25%, (*n* = 15), *p* = 0.005, Supplemental Figure S2).

**Figure 4:**
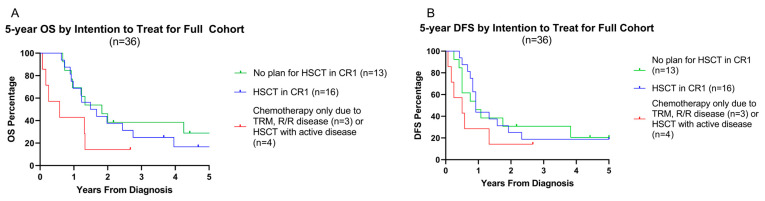
Five-year survivorship data of all patients by risk and intention to treat. (**A**,**B**) OS and DFS data, respectively, of all patients (*n* = 36) based on the intention to treat with allo-HSCT. Patients without a planned HSCT in CR1 included patients that had neutral or favorable cytogenetics and responded appropriately to therapy (green). This group includes patients that received chemotherapy only and never relapsed, as well as those who relapsed and subsequently received transplant in CR2. Patients who received chemotherapy only secondary to inability to undergo allo-HSCT due to uncontrolled disease, or those who received allo-HSCT with active disease, are included together as a high-risk patient population (red).

Additional survival analysis was conducted on the entire patient population identified (*n* = 36), including those excluded from cohort assignment. Five-year OS and DFS differed between groups when analyzed based on intention to treat with or without HSCT as part of upfront therapy (Figure 4A,B). Patients without a plan for HSCT in CR1 (OS: 36.4%, *p* = 0.07; DFS: 27.3%, *p* = 0.29) or who received HSCT in CR1 (OS: 18.2%, *p* = 0.10; DFS: 18.8%, *p* = 0.29) had a trend towards better 5-year outcomes compared to those with relapsed/refractory disease preventing HSCT or those who received HSCT with active disease (OS: 14.3%, DFS: 14.3%).

### 3.5. Relapse- and Treatment-Related Mortality

**Figure 5:**
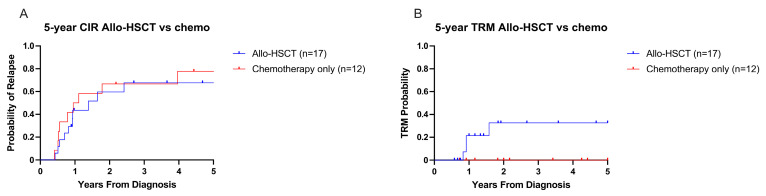
Five-year cumulative incidence of relapse- and treatment-related mortality of intention to treat cohorts. (**A**,**B**) Comparison of 5-year CIR and TRM, respectively, between the chemotherapy only vs. allo-HSCT groups.

Relapse was the primary cause of death in this cohort, with a 5-year CIR of 0.78 (Figure 5A) with no significant difference between groups (CIR: 0.78 for chemotherapy alone vs. 0.68 for chemotherapy + allo-HSCT, *p* = 0.63, Figure 5A). Median time from diagnosis to relapse was nine months (range: 3–48 months). Isolated BM relapse (*n* = 11) was more common than combined CNS and BM relapse (*n* = 2). Patients who relapsed received a median of two different salvage chemotherapy regimens (range: 0–7). Five-year cumulative incidence of TRM was 0.13 in the entire cohort (Figure 5B). There was an observed difference in TRM between groups, with a trend to higher TRM for patients receiving chemotherapy + allo-HSCT (0.33 vs. 0), though this was not statistically significant (*p* = 0.07, Figure 5B). All but one of the TRM-related deaths were due to infection. Two deaths secondary to infection occurred prior to the third cycle of chemotherapy and were not included in the intention to treat comparison groups due to the deaths occurring prior to treatment divergence of the groups.

### 3.6. Influence of Pre-HSCT Disease Status on Overall and Disease-Free Survival

**Figure 6:**
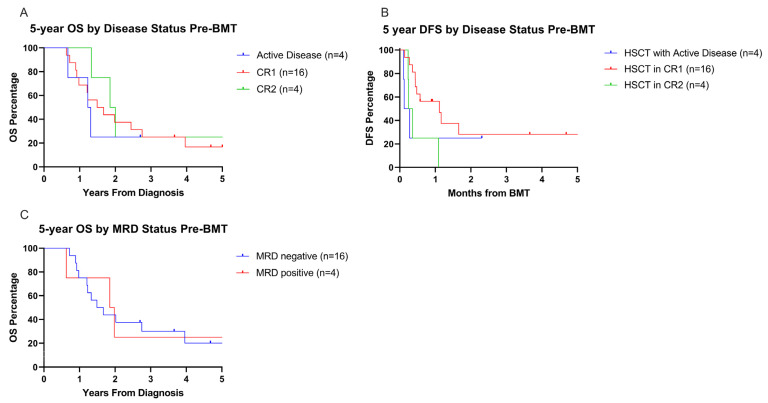
Five-year OS and DFS outcomes after transplant based on pre-BMT disease and MRD status. (**A**,**B**) OS and DFS by disease status prior to the first transplant, with DFS measured starting from time of transplant. (**C**) Five-year OS based on the MRD status prior to first transplant, excluding patients with active disease. MRD positivity defined as disease >0.1% myeloid blasts detected.

Five-year OS was low regardless of pre-HSCT disease status (5-year OS: CR1 = 16.7%, *n* = 16; CR2 = 25%, *n* = 4; active disease = not reached, *n* = 4; *p* = 0.75, Figure 6A). Five-year DFS, measured from time of transplant, was higher for patients who received HSCT in CR1 (28.1%) compared to patients in CR2 (0%) prior to HSCT, though not statistically significant (*p* = 0.09, Figure 6B). Five-year OS and DFS for patients with active disease at transplant is not reportable as all patients died or were censored for loss to follow up prior to the five year mark. The detection of MRD prior to HSCT for patients in CR1 or CR2 did not impact 5-year OS (*p* = 0.94, Figure 6C).

### 3.7. Post-HSCT Outcomes

All patients had neutrophil engraftment post-HSCT (median day 19, range: 12–22 days). No patients experienced graft rejection or failure. Incidence of grade III–IV acute GVHD and extensive chronic GVHD was low (8% and 4%, respectively). Seventeen patients (71%) relapsed post-HSCT (median day 131 post-HSCT, range: 40–425 days). Eight of these relapsed patients underwent a second HSCT and all died (*n* = 6 from relapsed disease after 2nd HSCT, *n* = 2 from HSCT-related TRM while in remission). Two patients received a second HSCT while in CR for poor graft function and autoimmune hemolytic anemia, respectively. Both patients died, one from relapsed/progressive disease and the other one from TRM (disseminated adenovirus infection).

## 4. Discussion

Our study represents one of the largest single-center cohorts of pediatric patients with non-DS-AMKL, a rare AML subtype characterized by its dismal prognosis and high risk of relapse. In this population, OS and DFS were poor regardless of the incorporation of allo-HSCT in the treatment plan. We acknowledge that some of the patients designated to proceed to transplant in CR1 were inherently more high-risk compared to the chemo-only group by nature of their cytogenetics and response to induction. Therefore, transplant may have improved the overall survival of the high-risk subset of patients to more closely resemble the survival of the relatively more favorable group who received chemotherapy only. However, due to a small sample size and no control group, we cannot make any substantial conclusions on the role of transplant in patients with these high-risk cytogenetic features. A small number of patients lacking high-risk features (per COG AAML1031) achieved long-term survival with chemotherapy alone. Patients who received allo-HSCT frequently had relapses of their disease regardless of pre-HSCT remission or MRD status, leading to comparable OS in patients transplanted in CR1, CR2, or with active disease. The CIR for this cohort was higher than reported relapse rates in children diagnosed with non-DS-AMKL and treated with HSCT [21]. Relapse was the leading cause of death while TRM was low in both groups. A small group of patients underwent a second allo-HSCT after relapse; however, none of them survived and five out of eight relapsed, highlighting the high incidence of relapse even after a second HSCT.

Initial response to induction chemotherapy with achievement of CR has been identified as an important prognostic factor for pediatric non-DS-AMKL [3]. However, attempts to intensify induction chemotherapy have had mixed outcomes and persistently lower OS for pediatric non-DS-AMKL when compared to other AML subtypes [10,15,16]. Chisolm et al. reviewed COG AMKL patient data from AAML 0531 and 1031 and showed that the former had better 5-year EFS and OS outcomes (5-year EFS of 62 ± 14% vs. 37 ± 13%, *p* = 0.009, respectively; 5-year OS of 64 ± 14% vs. 45 ± 14%, *p* = 0.069, respectively), indicating the relative success of the incorporation of gemtuzumab ozogamicin compared to bortezomib, as well as the necessity for five cycles of therapy for higher risk patients [12]. Our cohort was consistent with prior reports suggesting that 50–80% of patients achieve an MRD CR after induction chemotherapy [1,3,10,15,16,22]. Notably, a European group reported higher CR rates among the AML-BFM 87, AML-BFM 93, and AML-BFM 97 trials (58%, 77%, and 84%, respectively) with the use of increased cumulative dosage of cytarabine and anthracyclines during induction [16]. Conversely, reduced cytarabine exposure in low-risk AMKL patients led to lower disease-free and overall survival [23]. However, partial- and non-response remained higher in the non-DS-AMKL population compared to other AML types, and outcomes remained poor in non-responders, regardless of the use of HSCT. In our cohort, seven out of twelve patients treated with chemotherapy only developed refractory or relapsed disease that did not allow them to proceed to allo-HSCT, leading to their death. In contrast, two out of three of the survivors in the chemotherapy-only group achieved MRD-CR after induction I chemotherapy, with the third achieving MRD- CR after induction II. This observation highlights the significant impact of lack of initial response to chemotherapy on survival outcomes, emphasizing the critical importance of effective up-front treatment.

Non-DS-AMKL is often characterized by a complex and heterogeneous cytogenetic profile with prognostic implications [2,6,18]. This was reflected in our cohort, as we identified loss of 7q and monosomy 7, KMT2A rearrangements, complex karyotypes, and several recurrent genetic alterations including CBFA2T3::GLIS2 fusions (*n* = 4), RBM15::MLK1 fusions (*n* = 1), and GATA2 mutations (*n* = 1). The presence of RBM15(OTT)::MKL1(MAL) fusions has been associated with better prognosis in patients treated with chemotherapy only [1], although the only patient with this fusion in our cohort died from refractory disease and the three survivors in the chemotherapy-only group in our cohort did not support this finding. Translocations involving NUP98::KDM5A, CBFA2T3::GLIS2 fusions, KMT2A-rearrangements, and monosomy 7 are associated with a worse prognosis and lower OS, EFS, and RFS, regardless of other factors or variables [12,18,24]. It is well known that patients with CBFA2T3:GLIS2 fusions have an aggressive disease subtype with poor outcomes [7]. We did not examine the relationship between cytogenetic subgroups or distinct genomic alterations and outcomes in our cohort due to insufficient sample size. Given recent advances identifying high-risk genetic alterations and cytogenetics in AML, further investigation of their impact on outcomes is warranted in larger, prospective studies.

Data clarifying the role of allo-HSCT in pediatric patients with non-DS-AMKL is sparse. While some groups have argued that allo-HSCT in CR1 is beneficial compared to chemotherapy alone [3,8], outcomes remain poor, with high incidence of relapse in the first year post-HSCT. Athale et al. reported improved 2-year OS (30% vs. 0%, *p* < 0.001) and EFS (26% vs. 0%, *p* = 0.019) in patients who received HSCT in CR1 compared to chemotherapy alone. In our cohort, allo-HSCT did not result in improved 5-year OS or DFS. This is related to the high incidence of relapse post-HSCT and reflects the failure of graft-versus-leukemia effect, salvage chemotherapy regimens, and second HSCT to induce durable disease remission.

In line with our findings, others have also failed to prove that incorporating allo-HSCT into the treatment of non-DS-AMKL improves outcomes [15,16,25]. The largest retrospective study published to date, evaluating the long-term outcomes of 203 pediatric non-DS AMKL patients after HSCT between 1986 and 2015, demonstrated 5-year OS and EFS rates of 43% and 38%, respectively, with a 5-year CIR of 53% [14]. Another recent, retrospective, single-institution study of 44 pediatric non-DS-AMKL patients treated with allo-HSCT from 1986 to 2016 reported 3-year OS ranging from 26% to 41% due to the high incidence of relapse in the first year post-HSCT [26]. They also observed a relationship between CR1 status at the time of transplant with decreased CIR [3], highlighting the grim outcomes of patients with persistent disease at the time of HSCT. Although this group recommends that allo-HSCT should be offered to patients with non-DS-AMKL in CR1, they acknowledge that if post-HSCT relapse occurs, salvage is unlikely. Interestingly, non-Hispanic Caucasian race was identified as a good prognostic factor in their study. Our patient population was mostly Hispanic and African American, with a low percentage of non-Hispanic White or Caucasian patients. We did not investigate whether race or ethnicity had a substantial effect in clinical outcomes in our cohort and published data are based on small patient numbers. Several studies have investigated this question, with mixed data regarding the correlation of ethnicity, race, or socioeconomic status with the outcome of HSCT [27]. However, disparities in outcomes of AML patients treated with standard chemotherapy are repeatedly documented in the literature [28,29]. This warrants additional investigation to determine if certain racial or ethnic backgrounds represent higher risk populations due to the contribution of differences in disease biology, social, economic, and cultural factors leading to racial-related outcome disparities.

In this cohort, treatment-related mortality was low and largely due to infectious complications. Two patients died due to bacterial sepsis and respiratory failure while receiving induction chemotherapy, which highlights the low but grave risk of fatal infections while receiving intensive chemotherapy. All but one of the patients who died from infection post-HSCT had viral infections after day 100 from HSCT. Transplant-associated complications were infrequent and did not contribute to significant mortality in our cohort. We report low TRM in both groups, but most importantly, in patients that received chemotherapy followed by allo-HSCT, as reported by other groups [30]. Although HSCT did not improve outcomes, mortality related to transplant-associated complications such as graft rejection, failure, and acute and chronic GVHD did not play a role in the poor survival outcomes observed. This highlights the significant advancements made over recent decades in the management of post-transplant complications.

## 5. Conclusions

In conclusion, non-DS-AMKL represents an aggressive AML sub-type, with poor outcomes compared to other AML types, despite the use of allo-HSCT after achieving CR1 with intensive initial chemotherapy. In this cohort, the intent to treat with up-front allo-HSCT in CR1 was generally equivalent to chemotherapy alone when evaluating 1 and 5-year OS due to the high rate of relapse in both groups. This suggests the need for post-transplant maintenance strategies to help prevent relapse and improve survival. Although this report represents one of the largest single-institution studies of non-DS AMKL outcomes, the small sample size, due to the rarity of this disease, limited our ability to detect a difference in outcomes by treatment approach. Cooperative studies are needed to further understand the impacts of disease biology, cytogenetics, and genetic alterations on overall outcomes. Novel approaches to induction therapy, targeted agents, and agents with more effective BM penetration are urgently needed to achieve durable remission in non-DS-AMKL; for example, immunotherapy [31], Menin inhibitors [32], and FOLR1-directed therapies [33] are promising approaches being studied to address the current dismal outcomes of this disease.

## Figures and Tables

**Table 1:** Patient and disease characteristics.

Patient and Disease Characteristics—By Intention to Treat			
	Chemotherapy Alone *n* = 12	Chemotherapy and Allo-HSCT (*n* = 17)	*p*-Value
**Gender**	**Number (%)**	**Number (%)**	0.67
Male	4 (33)	7 (41)	
Female	8 (67)	10 (59)	
**Self-Described Race/Ethnicity**			0.13
Hispanic White	8 (67)	4 (24)	
Non-Hispanic African American	3 (25)	7 (41)	
Non-Hispanic White	1 (8)	4 (24)	
Other *	0	2 (12)	
**Age At Diagnosis (years)**			0.60
Median	2	2	
Range	0.7–17	0.6–15	
0 to 5 years	10 (75)	13 (76)	
6 to 10 years	0	2 (12)	
11 to 15 years	1 (8)	2 (12)	
>15 years	1 (8)	0	
**CNS Disease Status at Diagnosis**			0.25
Positive	1 (8)	4 (24)	
Negative	11 (92)	12 (71)	
Unknown	0	1 (6)	
**Cytogenetics**			0.09
Monosomy 7	0	2 (12)	
KMT2A-rearranged	1 (8)	5 (29)	
CBFA2T3::GLIS2 fusion	1 (8)	3 (18)	
Normal	3 (25)	0	
Other	7 (58)	6 (35)	
Not available	0	1 (6)	
**Risk Stratification**			0.015
Low Risk	9 (75)	5 (29)	
High Risk	3 (25)	12 (71)	
Initial Chemotherapy Regimen **			0.38
POG 9421	1 (8)	4 (24)	
AML 2002	1 (8)	2 (12)	
AAML0531	5 (42)	2 (12)	
AAML1031	5 (42)	8 (47)	
AAML1831	0	1 (6)	

* Other = Asian, Non-Hispanic American Indian. ** 14 patients were treated according to the corresponding protocol; 15 patients were enrolled on study.

**Table 2:** Patient and transplant characteristics—patients treated with chemotherapy and Allo-HSCT.

Patient and Transplant Characteristics for Patients Treated with Chemotherapy and Allo-HSCT, *n* = 24				
**Disease status before allo-HSCT**	**Number (%)**		**Conditioning regimen**	**Number (%)**
CR 1	16 (67)		Busulfan/Cyclophosphamide based ^^^	12 (50)
CR2	4 (16)		Cytarabine/Cyclophosphamide based ^&^	7 (29)
Active	4 (16)		Fludarabine/Cyclophosphamide	2 (8)
**MRD status by flow cytometry before allo-HSCT (*n* = 20)**			Other ^#^	3 (13)
Positive	4 (20)		**Relapsed after allo-HSCT**	
Negative	16 (80)		Yes	17 (71)
**Year of allo-HSCT**			No	7 (29)
2000–2009	9 (38)		**Post-HSCT day at relapse**	
2010–2022	15 (62)		Median	131
**Age at time of allo-HSCT (years)**			Range	40–425
Median	3		**Acute graft-versus-host disease grade**	
Range	0.8–19		None	18 (75)
**Time from diagnosis to allo-HSCT (months)**			Grade I–II	3 (13)
Median	5		Grade III–IV	2 (8)
Range	2.7–23.2		Unknown	1 (4)
**Indication for allo-HSCT**			**Chronic graft-versus-host disease**	
Induction failure *	3 (13)		None	22 (92)
Considered high-risk disease	8 (33)		Limited	0
MRD+ disease after induction I/II	8 (33)		Extensive	1 (4)
Relapsed disease after chemotherapy only	5 (21)		Unknown	1 (4)
**Stem Cell Source**			**Censored for Second Allo-HSCT**	
Bone marrow	15 (62)		Yes	9 (38)
Peripheral blood stem cells **	5 (21)		No	15 (62)
Umbilical cord blood	3 (13)		**Indication for Second Allo-HSCT**	
Unavailable	1 (4)		Post-HSCT Relapse	7 (78)
**Donor Type**			Poor Graft Function	2 (22)
Matched sibling donor	4 (16)			
Matched unrelated donor	9 (38)			
Mismatched unrelated donor	2 (8)			
Haploidentical	9 (38)			

* Patients with ≥5% myeloid blasts detected in the bone marrow by morphology at the end of induction I chemotherapy. ** *n* = 3 received CD34-selected PBSCs. ^ Includes Busulfan/Cyclophosphamide(*n* = 4), Busulfan/Cyclophosphamide/Campath (*n* = 6), and Busulfan/Cyclophosphamide/Fludarabine (*n* = 4). & Includes Cytarabine/Cyclophosphamide/TBI/Campath (*n* = 6). # Includes Busulfan/Fludarabine/ATG, Busulfan/Fludarabine/Campath, and Busulfan/Thiotepa/Fludarabine/ATG/Rituximab, all *n* = 1.

## Data Availability

The data presented in this article are available from the corresponding author on request due to necessity to protect patient privacy.

## Supplementary Materials

The following supporting information can be downloaded at https://www.mdpi.com/article/10.3390/cancers17213511/s1, Figure S1: One-year OS allo-HSCT vs. chemotherapy only; Figure S2: Five-year OS based on year of HSCT.
