# Supplementary material for: Pediatric Non-Down Syndrome Acute Megakaryoblastic Leukemia Patients Have Dismal Outcomes Irrespective of Allogeneic Hematopoietic Stem Cell Transplant: A Single-Center Experience
Source: Cancers (Basel). 2025 Oct 31;17(21):3511. doi: 10.3390/cancers17213511 (PMC12609084; doi:10.3390/cancers17213511)

Supplemental Figure S1

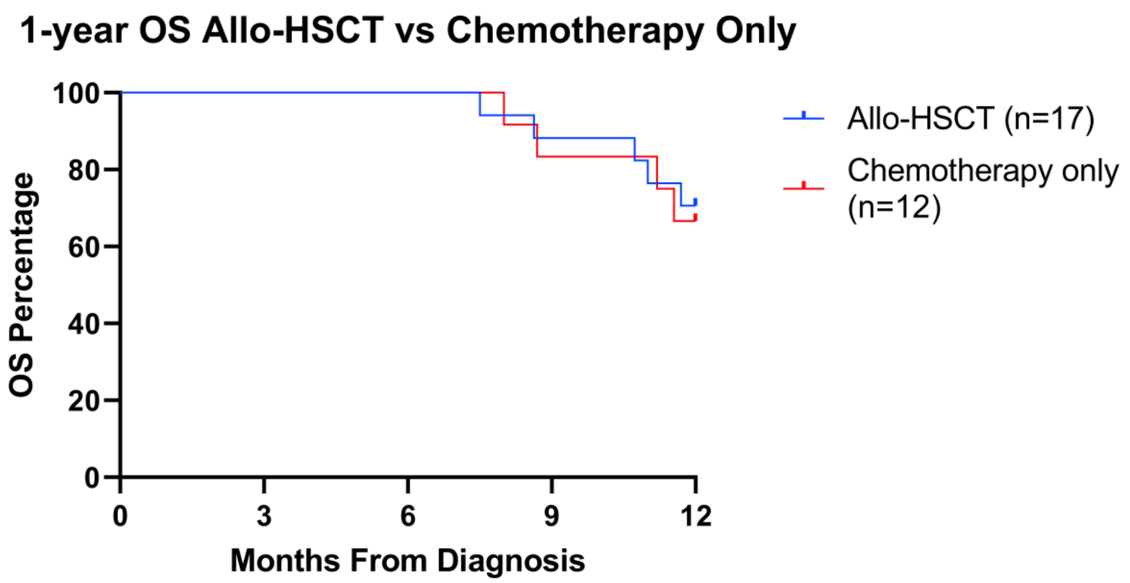

Supplemental Figure S2

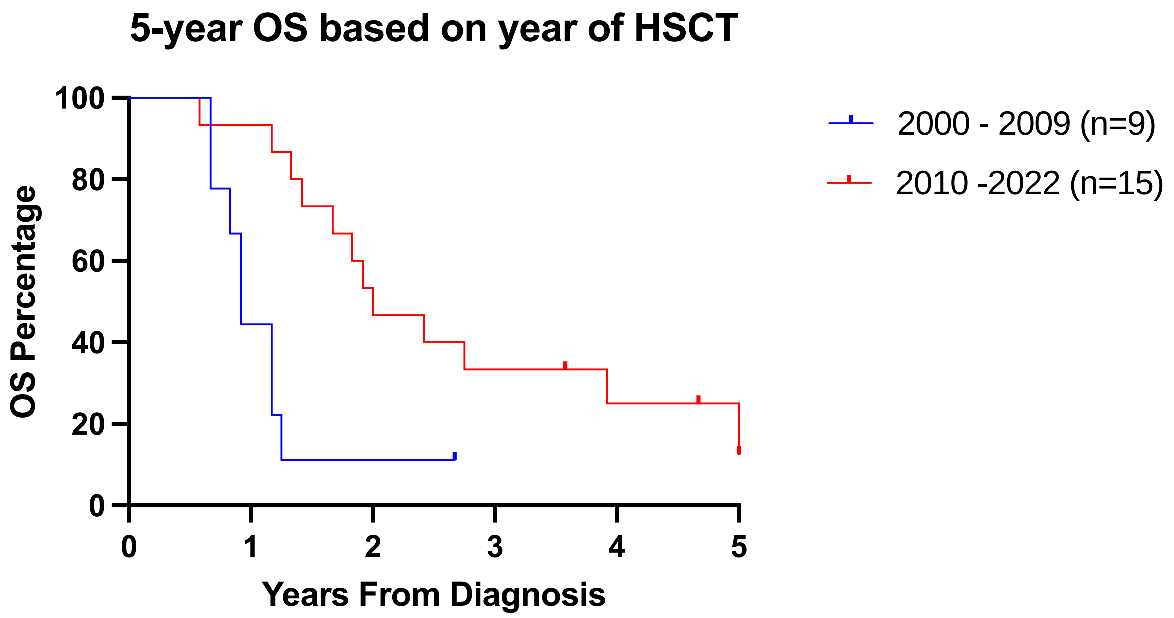

Supplement: Supplementary file 1 [file cancers-17-03511-s001.zip › cancers-3937741-supplementary.pdf]
